# Stable serum peptidoglycan fragment levels do not support leaky gut in the acute phase or at one month following “mild” traumatic brain injury: A preliminary study

**DOI:** 10.1016/j.bbih.2026.101192

**Published:** 2026-02-05

**Authors:** Koen Visser, Yue Wang, Jon D. Laman, Myrthe E. de Koning, Xiaoli Xu, Andrei A. Vakhtin, Andrew R. Mayer, Daan Kremer, Stephan J.L. Bakker, Harry van Goor, Joukje van der Naalt, Arno R. Bourgonje, Harm J. van der Horn

**Affiliations:** a Department of Neurology, University of Groningen, University Medical Center Groningen, Groningen, the Netherlands; b A∗STAR Infectious Diseases Labs, Agency for Science, Technology and Research (A∗STAR), Singapore; c Section of Medical Biology of the Department of Pathology and Medical Biology, University of Groningen, University Medical Center Groningen, Groningen, the Netherlands; d Department of Neurology, Medical Spectrum Twente, Enschede, the Netherlands; e The Mind Research Network; Lovelace Biomedical and Environmental Research Institute, Albuquerque, NM, USA; f Department of Internal Medicine, Division of Nephrology, University of Groningen, University Medical Center Groningen, Groningen, the Netherlands; g Division of Pathology of the Department of Pathology and Medical Biology, University of Groningen, University Medical Center Groningen, Groningen, the Netherlands; h The Dr. Henry D. Janowitz Division of Gastroenterology, Department of Medicine, Icahn School of Medicine at Mount Sinai, New York, NY, USA; i Department of Gastroenterology and Hepatology, University of Groningen, University Medical Center Groningen, Groningen, the Netherlands

**Keywords:** Concussion, Gut permeability, Microbiome, Microbiota, Peptidoglycan, Toll-like receptor

## Abstract

The gut-brain axis is increasingly recognized as contributor to the pathophysiology of brain disorders, in part through its influence on inflammation. Impaired gut health can lead to so-called *leaky gut,* allowing bacterial cell wall fragments such as peptidoglycan-derived muramyl dipeptide (MDP) to translocate to the circulation. MDP can activate microglia, key mediators of neuroinflammation, via the intracellular receptor nucleotide-binding oligomerization domain-containing protein 2 (NOD2). Although neuroinflammation is a hallmark of “mild” traumatic brain injury (mTBI), the link between leaky gut and mTBI remains largely unexplored.

This preliminary prospective study investigated whether mTBI leads to increased MDP levels and NOD2 activation in the acute phase (N = 246; median 106 min post-injury) and at a ∼1-month follow-up visit (N = 140; median 32 days) in an emergency department cohort, relative to healthy controls (HC; N = 31, with N = 26 at ∼1-month). Serum MDP concentration was measured using an indirect competitive ELISA. Engagement of the pro-inflammatory nuclear factor (NF)-κB pathway was measured in NOD2-transfected cells. Additionally, blood interleukin (IL)-6 and IL-10 levels were quantified. Clinical outcome was measured at six months post-injury using the extended Glasgow Outcome Scale and a symptom questionnaire.

Linear mixed effects models showed that concentrations of MDP remained stable across visits in both mTBI and HC (*P* = 0.62), with no significant main effect of group (*P* = 0.16) or group × visit interaction (*P* = 0.25). In contrast, for engagement of NF-κB signaling in NOD2-expressing cells, a significant group × visit interaction (*P* = 0.004) was observed, with an elevation in mTBI relative to HC at ∼1-month post-injury (*P* = 0.01, Cohen's *d* = 0.48), but not in the acute phase (*P* = 0.22, *d* = 0.22). This elevation was associated with higher IL-6 (β = 0.16, *P* = 0.02) and IL-10 (β = 0.17, *P* = 0.006) levels in the acute phase. No associations with clinical outcome were observed.

In conclusion, our preliminary null findings for serum MDP do not directly support the emergence of leaky gut in either the acute phase or at ∼1-month following mTBI. However, transient increases in MDP occurring during the first month cannot be ruled out based on our findings. Increased engagement of the NF-κB pathway in NOD2-expressing cells likely reflects (damage-associated) mechanisms other than MDP. Assessment of NF-κB signaling may serve as a useful marker for studying chronic (neuro)inflammation following mTBI, complementing interleukin responses in the acute phase, an avenue warranting further investigation.

## Highlights

• Serum peptidoglycan fragment levels did not change <24 h or ∼1-month post mild TBI.
• The innate immune receptor NOD2 in microglia binds peptidoglycan fragments.
• We quantified NF-κB activity within human embryonic kidney cells expressing NOD2.
• ∼1-month post-injury serum increased NF-κB activity in these cells.
• This likely reflects peptidoglycan-independent neuroinflammatory mechanisms.

## 1 Introduction

While the terminology is seemingly innocuous, “mild” traumatic brain injury (mTBI) poses a significant public health burden (Maas et al., 2022; van der Horn et al., 2020; van der Naalt et al., 2017). Most patients will fully recover without specific treatment, yet a substantial subset of patients (estimated 30-50%) experiences residual symptoms lasting months to years, affecting quality of life and socioeconomic participation (Cassidy et al., 2014; McMahon et al., 2014), and questioning current nomenclature used to rate “mild” injury severity (Manley et al., 2025). The persistence of symptoms after mTBI is likely precipitated by a complex interplay between acute injury and secondary biochemical injury mechanisms together with pre-existent psychological factors (Sage et al., 2022; van der Horn et al., 2020; van der Naalt et al., 2017). Key secondary injury processes comprise neuroinflammation, oxidative distress and cellular energy disturbances, occurring at varying intensities, durations, and time points across individual patients (Giza and Hovda, 2014; Maas et al., 2022). In alignment with the recently proposed CBI-M framework (clinical, biomarkers, imaging, and modifiers), a better understanding of these secondary injury processes may reveal early biomarkers that facilitate more precise characterization of individual patients (Manley et al., 2025). This may include their risk for poor outcomes and their potential eligibility for (novel) targeted treatments. Importantly, interactions with organs outside the brain may modulate these secondary injury processes. The gut–brain axis is a notable example that is increasingly implicated in the pathophysiology of brain disorders, including TBI (Aghakhani, 2022; Munley et al., 2023; Oyovwi and Udi, 2024; Panther et al., 2022; Sundman et al., 2017).

Dysbiosis is defined as a change in the composition of gut microorganisms relative to healthy individuals (Petersen and Round, 2014). This condition is associated with local gut inflammation that disrupts intestinal barrier integrity, a process often referred to as leaky gut in popular context (Hang et al., 2003; Kelly et al., 2015; Pan et al., 2019). Increased permeability of the gut allows for leakage of bacterial cell membrane fragments into the circulation, where they may initiate or modulate inflammatory responses, for example through (further) activation of microglia, the brain's primary immune cells involved in neuroinflammation (Arrieta et al., 2006; Birg et al., 2024; Kelly et al., 2015; Mourits et al., 2020; Olsen et al., 2013; Oyovwi and Udi, 2024). Furthermore, these fragments may (further) disrupt blood brain barrier (BBB) integrity (Braniste et al., 2014; Laman et al., 2020; Parker et al., 2020). Therefore, leaky gut may directly affect the secondary injury cascade of TBI (Oyovwi and Udi, 2024; Sundman et al., 2017). Most evidence on the gut-brain axis in TBI stems from animal studies and studies on moderate-to-severe TBI in humans (Aghakhani, 2022; Hang et al., 2003; Nicholson et al., 2019; Sundman et al., 2017), with mixed results, particularly regarding changes in specific microbiota communities. Limited knowledge exists regarding dysbiosis and gut permeability in mTBI (Aghakhani, 2022). To our knowledge, only one human study on four patients with sports-related concussion found indications for dysbiosis (Soriano et al., 2022).

Therefore, the current preliminary study set out to explore leaky gut following mTBI by measuring bacterial cell wall fragments in the bloodstream. We focused on bacterial peptidoglycan, a complex polymer critical to the structure of all Gram-positive and Gram-negative bacteria. With an estimated 10–100 g of peptidoglycan present in the gut microbiota of healthy adults, it likely represents the highest biomass of any bacterial molecule (Laman et al., 2020). Muramyl dipeptide (MDP) is a small peptidoglycan fragment that can leak into the circulation and is a canonical binder of nucleotide-binding oligomerization domain-containing protein 2 (NOD2) expressed intracellularly by innate immune cells, including microglia (Huang et al., 2019; Spielbauer et al., 2024). Binding this pattern recognition receptor initiates inflammation through the pro-inflammatory transcription factor Nuclear Factor κB (NF-κB) (Negroni et al., 2018).

We measured both MDP concentrations and NOD2 activity in serum collected during the acute phase (<24 h) and at ∼1-month after mTBI and compared these to healthy controls (HC) assessed at equivalent time intervals. We hypothesized that patients with mTBI would show increased serum concentrations of MDP and associated NOD2 activation in the acute phase, returning to HC levels at one-month follow-up. Furthermore, we expected these changes to be related to systemic inflammation, as reflected by elevated interleukin (IL)-6 and IL-10, which we previously found to be elevated in the acute phase (Visser et al., 2024a).

## 2 Methods

### 2.1 Participants

**Fig. 1:**
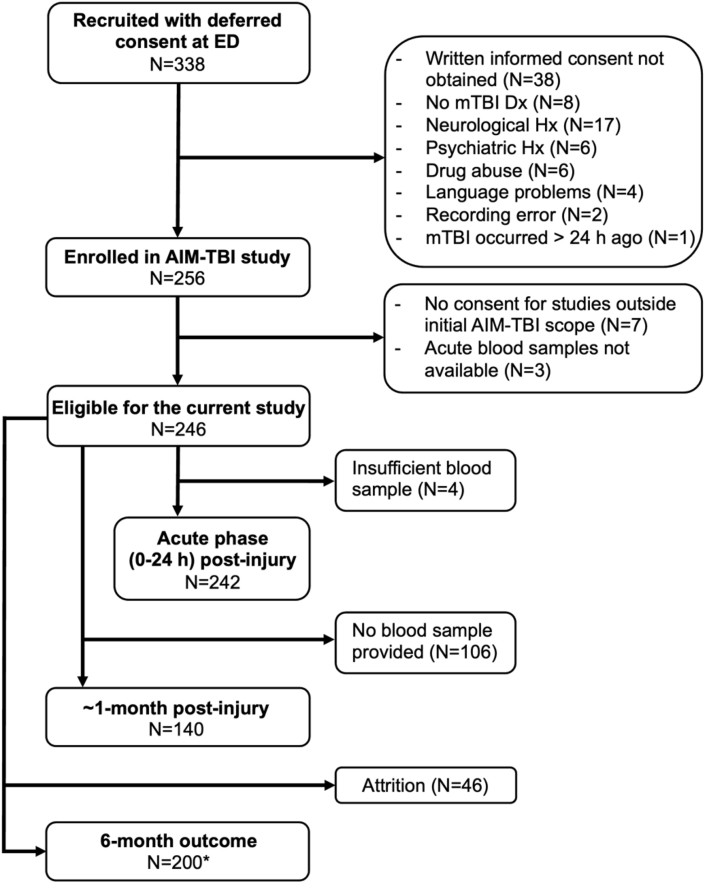
Patient enrollment flowchart. ∗Retention rate based on patients who returned the Glasgow Outcome Scale Extended (GOS-E; N = 200, 81%). *Abbreviations*: Dx, diagnosis; ED, emergency department; GOS-E, Glasgow Outcome Scale Extended; Hx, history; mTBI, mild traumatic brain injury.

The current study was performed within the AIM-TBI study framework, complementing its initial scope (Dutch trial register no. NL8484). A detailed description of the AIM-TBI study including inclusion and exclusion criteria has been previously published (Visser et al., 2024a). Fig. 1 shows the patient enrollment flowchart for the current study. The final study sample consisted of 246 patients with mTBI, with 140 (56.9%) returning for a second blood draw at ∼1-month post-injury. The second time point was chosen because it aligns with the typical outpatient follow-up visit for patients who were admitted for their mTBI or who experience persistent symptoms. In addition, a group of 31 HC (N = 26 at ∼1-month follow-up, with one subject only providing a sample at follow-up) were recruited via word of mouth or flyers in the local community. This control group was recruited as part of an MRI sub-study (Visser et al., 2024b) and therefore not optimally age-matched with the total patient cohort.

The AIM-TBI study was approved by the Medical Ethical Committee of the University Medical Center Groningen (UMCG; METc, 2018/681), and all participants provided written informed consent. All study procedures were performed in accordance with the Declaration of Helsinki.

### 2.2 Acute injury measures

All patients underwent head computed tomography (CT) scans at the ED. The injury severity score (ISS) and abbreviated injury score (AIS) were used to assess general trauma severity (Baker et al., 1974). Extracranial injury was computed from ISS and AIS sub-scores as a dichotomous variable (i.e., mTBI with no or only superficial extracranial injury, such as skin abrasions vs. mTBI with additional extracranial injury, such as a fracture) based on the approach by Chaban et al. (2020). Additional injury factors such the presence of alcohol intoxication and prior TBI were also recorded.

### 2.3 Clinical outcome measures

At six months post-injury, clinical outcomes were assessed using questionnaires sent either digitally via email through REDCap or by regular mail. Six months was chosen as the time point for long-term outcome assessment as most patients are expected to have resumed work and other daily activities by then, and it aligns with our previous research on mTBI (van der Naalt et al., 2017). Post-traumatic symptoms were measured using the 21-item Head Injury Symptom Checklist (HISC) (de Koning et al., 2016). Total pre-to postinjury increase in number of symptoms was used for further analyses. Functional recovery was determined using the Glasgow Outcome Scale-Extended (GOS-E) (Wilson et al., 2021). These scores were dichotomized for further analyses into complete recovery (GOS-E = 8) and incomplete recovery (GOS-E < 8). In cases where patients did not return their questionnaires, additional steps were taken to at least collect GOS-E data, such as reminder calls or administering this assessment by telephone upon request.

### 2.4 Blood sample collection and preparation

Whole blood was collected using 1 × 10 mL BD vacutainer (BD, Plymouth, UK) and 1 × 10 mL EDTA vacutainer and sent to the laboratory immediately after collection. Samples for serum were allowed to clot for 60 min after collection and then centrifuged at room temperature at 1300*×g* for 10 min. To obtain plasma, samples were centrifuged immediately at 1300*×g* for 10 min. Serum and plasma were aliquoted and stored at −80 °C. All samples used in the current study had not undergone a previous freeze-thaw cycle. Sample storage duration varied according to the date of inclusion, with a maximum difference of approximately three years (study inclusion between January 2020 and December 2022). Control subjects were recruited throughout the same period, resulting in natural temporal interspersion of groups and minimizing the risk of systematic group-related bias. Serum samples were shipped on dry ice to Singapore for analyses of MDP and NOD2. It was confirmed that all samples remained frozen upon arrival. Plasma samples were used for inflammatory analyses (IL-6 and IL-10) performed at the UMCG.

### 2.5 Quantification of MDP

Indirect competitive enzyme-linked immunosorbent assay (icELISA) was used for serum MDP quantification, as described previously (Huang et al., 2019; Mourits et al., 2020; Tan et al., 2021). Reagents used in the icELISA included the anti-MDP monoclonal antibody 2E7, human serum albumin (HSA)–MDP conjugates, horseradish peroxidase (HRP)-conjugated anti-mouse antibody (GE Healthcare), MDP stock solutions, hydrogen peroxide (VWR International), and 2 M sulfuric acid. icELISA was performed in 96-well half-area plates (Costar; Corning). Absorbance was measured at 450 nm using a plate reader (Tecan Infinite 200 Pro; Tecan Life Sciences). A 2-fold serial dilution of authentic MDP was included on each plate to generate a standard curve for calculating the amount of MDP in each sample on the same plate. This approach largely eliminates variability arising from differences between sample batches, assays performed on different days, or sample handling. Samples were distributed across assay plates in a pseudo-random manner based on order of patient/control inclusion, with longitudinal samples from each subject analyzed on the same plate. Laboratory personnel were blinded to group labels during sample analysis. Further details can be found in the Supplementary Materials.

### 2.6 NOD2 activation assay

NOD2 activation was measured using human embryonic kidney (HEK)-Blue™-hNOD2 cells that stably co-express human NOD2 along with NF-κB-inducible secreted embryonic alkaline phosphatase (SEAP) reporter gene (Invivogen). HEK-Blue™-hNOD2 cells detect stimulants of the human NOD2 receptor by induction of SEAP. The levels of SEAP were measured with HEK-Blue™ Detection (InvivoGen), a cell culture medium that is designed for real-time detection of SEAP. Cells were cultured and maintained according to the manufacturer's recommendations. To evaluate NOD2 activation in serum, 10 μL of serum was added to a 96-well plate with 90 μL of hNOD2 cells (approximately 250,000 cells/mL). NF-κB-induced SEAP activity was assessed in the culture supernatant using HEK-Blue™ and read at optical density 650 nm after 16 h. A standard curve was generated using twofold serial dilutions of standard MDP (and therefore NOD2 activity levels are expressed in ng/mL). Average intra-assay variability was <2%.

### 2.7 Analysis of systemic inflammatory markers

Plasma concentrations of IL-6 and IL-10 were measured using a custom V-PLEX® assay (Meso Scale Discovery [MSD], Meso Scale Diagnostics, Rockville, MD). This selection of cytokines was based on a systemic review (Visser et al., 2022). Preparation of assays was performed according to manufacturer's instructions. Reading of plates was performed on an MSD QuickPlex SQ 120 MM instrument. Final concentrations were determined from a 4-parameter logistic curve that was fitted to calibrator signals using the MSD Discovery Workbench software 4.0. The lower limit of detection (LLOD) for IL-6 and IL-10 were 0.06 pg/mL and 0.51 pg/mL, respectively. Values below the LLOD (one mTBI and one HC for IL6; one mTBI for IL10) were replaced by the LLOD.

Only acute (and not ∼1-month) IL-6 and IL-10 concentrations were used for statistical analyses, as in our previous study on an almost identical cohort we found that these markers were increased (predominantly) in the acute phase after mTBI (Visser et al., 2024a).

### 2.8 Statistical analyses

Statistical analyses were performed in Python v3.9 using: Scipy (v1.13.1), Statsmodels (v0.14.4), Pymer4 (v0.8.2), Pandas (v2.2.2), and Numpy (v2.0.2). Boxplots with jittered points were generated using matplotlib (v3.4.2) and finalized in Inkscape (v1.0.2).

Normality of continuous variables was assessed visually using histograms and Q-Q plots and quantitatively using the Shapiro-Wilk test. Group differences in biological sex were evaluated with chi-square tests, while age differences were tested using the Mann-Whitney *U* test. MDP values were cube-root transformed and NOD2 activity data underwent log10 transformation to approximate normality. Outliers (1.5 × interquartile range [IQR] above third or below first quartile) were removed prior to statistical testing (MDP: 2 acute and 0 at ∼1-month; NOD 2: 5 acute and 1 at ∼1-month).

Two-way (group [mTBI vs. HC] × visit [acute vs. ∼1-month]) linear mixed models were conducted to examine the effects of mTBI on MDP and NOD2 activity levels. Age and sex were included as (nuisance) covariates. A random intercept for subject was included in all models. For NOD2 activity, an additional random intercept for plate was included to account for biological variation (of no interest) across batches of HEK cells as well as across plates. Type III sums of squares were used to test for main and interaction effects. Results were considered significant at a (two-tailed) Bonferroni corrected α = 0.05/2 = 0.025. In case of a significant group × visit interaction, post-hoc contrasts were used to decompose group effects at each visit separately. Cohen's *d* calculated from the marginal means of contrast tests were used as a measure of effect size. Additional sensitivity analyses were performed to compare acute MDP and NOD2 between patients who did and did not return for a second blood draw at ∼1-month. Furthermore, linear mixed models were repeated comparing HC with an optimally matched TBI subgroup. Further details regarding these analyses can be found in the Supplementary Materials.

In case of a significant main effect of group or a group × visit interaction, exploratory univariate linear regression analyses were conducted within the mTBI group to assess the association of MDP concentration and/or NOD2 activity (dependent variables) with acute inflammatory markers (IL-6 and IL-10 concentration; α = 0.05/2 = 0.025). Multivariate regression models were run at each visit to examine the association with acute injury variables (time to sample, extracranial injury [yes/no], alcohol intoxication [yes/no], lesions on CT [yes/no] and GCS), with inclusion of age, sex and (for NOD2 activity) plate as covariates. Wald tests were used to assess significance of model coefficients. Since data for prior mTBI [yes/no] was only available in 185/242 patients, this variable was examined separately using univariate regression.

Additional regression analyses were conducted to explore associations of MDP concentration and/or NOD2 activity (independent variables) with post-traumatic symptoms (HISC; linear model) and functional recovery (dichotomized GOS-E; logistic model) at six months post-injury (α = 0.05/2 = 0.025). Additional sensitivity analyses were performed to compare acute MDP and NOD2 data between patients who did and did not complete clinical outcome questionnaires (see Supplementary Materials for details).

## 3 Results

### 3.1 Participant characteristics

There was a significant difference in age between mTBI (median = 49, IQR = 33) and HC (median = 33, IQR = 17; *U* = 4842, *P* = 0.014). The distribution of biological sex was comparable between the mTBI and HC groups (χ^2^ = 0.478, *P* = 0.49). Data on injury and trauma care characteristics for the mTBI group are listed in Table 1.

**Table 1:** Injury and trauma care characteristics for patients with mTBI (N = 246).

Injury mechanism, n (%)	
*Motor vehicle accident*	54 (22%)
*Bicycle accident*	84 (34%)
*Falls*	86 (35%)
*Sports*	8 (3%)
*Other*	14 (6%)
GCS score, %	
*15*	61%
*14*	36%
*13*	3%
LOC, yes, n (%)	128 (52%)
PTA, yes, n (%)	180 (73%)
Presence of traumatic lesions on head CT, n (%)	58 (24%)
AIS head, median (IQR)	2 (2)
Presence of extracranial injury, n (%)	113 (46%)
Alcohol intoxication, yes, n (%)	56 (23%)
Time-to-acute-sample (min), median (IQR)	106 (122)
Interval injury to second blood draw (days), median (IQR)	32 (12)
Hospital admission, n (%)	126 (51%)
Previous mTBI, yes, n (%)	55 (30%)^a^

*Abbreviations:* AIS = abbreviated injury scale; CT = computed tomography; GCS = Glasgow coma scale score; ISS = injury severity score; LOC = loss of consciousness; mTBI = mild traumatic brain injury; PTA = post traumatic amnesia.

a Based on total N = 185 who provided information.

Regarding six-month outcome assessment in patients, completion rates were higher for the GOS-E (N = 200; 81%) compared to the HISC (N = 168; 68%). Approximately half of these patients (51%) had complete functional recovery (GOS-E = 8), and the average (±SD) post-traumatic symptom score was 5.1 (4.7).

### 3.2 MDP and NOD2 in mTBI versus HC

**Fig. 2:**
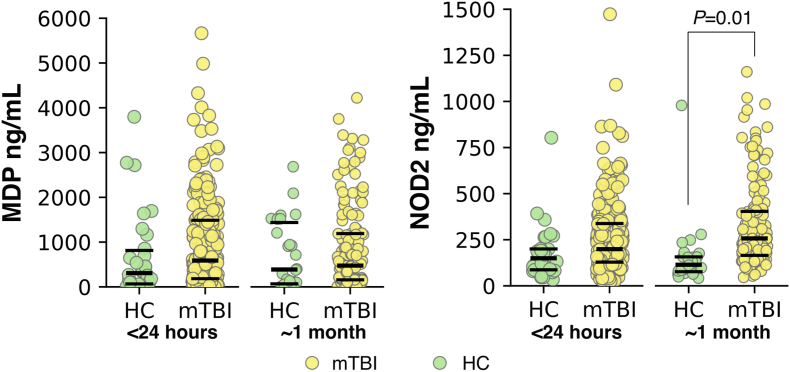
Plots showing (A) serum muramyl dipeptide (MDP) concentration and (B) nucleotide-binding oligomerization domain-containing protein 2 (NOD2) activity in patients with mild traumatic brain injury (mTBI) compared to healthy controls (HC) in both the acute phase (<24 h) and at ∼1-month post-injury. Lines represent medians with first and third quartiles. Note that, since the standard curve for the NOD2 assays was generated using standard MDP, NOD2 activity levels are expressed as ng/mL.

Linear mixed model analysis of MDP concentrations showed that the main effects of visit (*P* = 0.62) and group (*P* = 0.16), as well as the group × visit (*P* = 0.25) interaction, were not significant after accounting for age and sex (Fig. 2A). For NOD2 activity, a significant group × visit interaction was observed (*F*_1,1__73_ = 9.0, *P* = 0.004 < 0.025; Fig. 2B). Follow-up pairwise comparisons of estimated marginal means at each visit showed no significant difference between groups in the acute phase (*P* = 0.22, *d* = 0.22) but did demonstrate a significant increase for mTBI relative to HC at ∼1-month post-injury (*P* = 0.01, *d* = 0.48). Main effects of group (*P* = 0.049) and visit (*P* = 0.17) were not significant for NOD2 activity.

Sensitivity analyses comparing an optimally matched mTBI group to the HC group confirmed null findings for MDP (group: *P* = 0.91; group × visit: *P* = 0.25). For NOD2 activity, the group × visit interaction was no longer significant (*P* = 0.12). Instead, a main effect of group (mTBI > HC) with large effect sizes was observed (*P*_*acute*_ = 0.11, *d*_*acute*_ = 0.77; *P*_*subacute*_ = 0.02, *d*_*subacute*_ = 1.88), although this effect was not significant after correction for multiple comparisons (*P* = 0.038 > 0.025). Sensitivity analyses comparing patients who did and did not return for a follow-up blood draw at ∼1-month, did not show any significant group effects for acute MDP concentrations (*P* = 0.46) or NOD2 activity (*P* = 0.52).

### 3.3 Associations with IL-6 and IL-10 as markers of inflammation

Univariate regressions showed that concentrations of IL-6 (standardized *β* = 0.03, *P* = 0.60) and IL-10 (standardized *β* = 0.03, *P* = 0.48) in the acute phase were not associated with acute NOD2 activity in patients with mTBI. However, patients with higher levels of IL-6 (standardized *β* = 0.16, *P* = 0.02) and IL-10 (standardized *β* = 0.17, *P* = 0.006) in the acute phase had higher levels of NOD2 activity at 1-month post-injury.

### 3.4 Associations with acute injury variables

Multivariate regression showed that alcohol intoxication (N = 56; 23% of total mTBI) was associated with lower acute and ∼1-month NOD2 activity (Table S1). Furthermore, extracranial injury (N = 113; 46%) was associated with higher NOD2 activity at ∼1-month. Notably, the absence of associations between NOD2 activity and time to sampling suggests that temporal kinetics did not confound the main findings of our study. This also applies to MDP (see Table S2).

Separate univariate regression revealed that previous TBI (N = 55; 30%) was associated with higher NOD2 activity in the acute phase (standardized *β* = 0.3; *P* = 0.048).

### 3.5 Associations with clinical outcome

There were no significant associations of acute or one-month NOD2 activity with symptoms or functional recovery at six months post-injury (*P*'s > 0.05). Sensitivity analyses comparing patients who did and did not complete GOS-E or HISC did not show any significant group effects for acute MDP concentrations or NOD2 activity (all *P*'s > 0.05).

## 4 Discussion

In the current study, we aimed to explore the gut–brain axis following mTBI by measuring serum MDP, a peptidoglycan fragment found in bacterial cell walls and a potential indicator of gut permeability, as well as the activity of NOD2, an innate immune receptor triggered by MDP. Contrary to our initial hypothesis, we found no significant elevation of serum MDP concentrations in the acute phase following mTBI as compared to HC. Interestingly, a significant upregulation of NOD2 activity was found at ∼1-month post-injury rather than in the acute phase, which was significantly associated with the magnitude of IL-6 and IL-10 release in the acute phase. These findings suggest that NOD2 activity reflects chronic (neuro)inflammatory processes following mTBI that are independent of MDP. However, the possibility of a transient rise in MDP between the acute and ∼1-month post-injury assessments cannot be excluded based on the current data.

To our knowledge, serum MDP concentrations have not yet been investigated after TBI. Previous animal research on TBI has assessed the endotoxin lipopolysaccharide (LPS) (Hang et al., 2003), a fragment found in the cell wall of Gram-negative bacteria with potent ability to trigger the immune system in and outside of the brain (Birg et al., 2024; Zhao et al., 2019). In rats with severe TBI, LPS concentrations increase as early as 3 h post-injury, peaking at 72 h and declining after 7 days (Hang et al., 2003). In our study on “mild” TBI, however, most acute samples were collected within 3 h post-injury. This time point was chosen to align with previous biomarker studies in mild TBI, which commonly assess early biochemical responses within the first hours after injury. The second time point, at approximately 4–6 weeks post-injury, corresponds to the routine clinical follow-up in our outpatient setting and is frequently used in mTBI research to evaluate subacute recovery trajectories. We deem it possible that increases in systemic MDP concentration might have occurred in between these time points. This is further supported by another rodent study using a moderate TBI model, which demonstrated that the extent of dysbiosis peaked between day 2 and 3 post-injury, in a similar time-related fashion as MRI lesion volumes (Nicholson et al., 2019). It is therefore possible that our acute (median ∼2 h post-injury) and ∼1-month samples were respectively too early or late to detect alterations in gut barrier integrity, as these may have transiently peaked days post-injury and returned to baseline.

Although other peptidoglycan subunits are known, MDP is the canonical binder of NOD2 (Huang et al., 2019; Negroni et al., 2018). Therefore, the observation of persistently increased NOD2 activation at one-month post-injury with normal levels of MDP is likely explained by mechanisms independent from MDP. Potential candidate mechanisms include endoplasmic reticulum (ER) stress, which occurs when the capacity of the ER to fold proteins is overwhelmed. The resulting accumulation of unfolded proteins can trigger inflammation, in part through activation of NOD2 (Keestra-Gounder et al., 2016). Furthermore, NOD2 can be activated through sphingosine-1-phosphate, a lipid signaling molecule that is released during cellular stress (Pei et al., 2021) and plays a role in neuroinflammation after TBI (Cuzzocrea et al., 2018). Alternatively, our results may be explained by activation of Toll-like receptor (TLR)-3 and/or TLR-5, which are other pattern recognition receptors converging on the NF-κB pathway, and are still endogenously present on the HEK-NOD2 cells used in this study. The flagellin protein of the bacterial flagellum typically binds and activates TLR5 expressed by microglia. However, we consider it unlikely that the flagellin protein is released into the bloodstream, while MDP with low molecular weight and present at much higher biomass, would remain sequestered in the gut. Instead, damage associated molecular patterns (DAMPs), such as human host RNA released upon TBI, may trigger intracellular TLR3 (Cavassani et al., 2008; Krämer et al., 2022; Shi et al., 2019; Vourc'h et al., 2018). Future research with null (parental) or TLR HEK-Blue™ cells can shed more light on these mechanisms.

Notably, elevated concentrations of IL-6 and IL-10 in the acute phase were only related to NOD2 activation at one-month, and not the acute phase, post-injury. This suggests that the cytokine release by microglia and astrocytes precedes the systemic release of DAMPs capable of NF-κB activation but also implies that the magnitude of early inflammation is associated with the magnitude of more chronic inflammatory processes (Cavassani et al., 2008; Krämer et al., 2022; Visser et al., 2022). It is possible that the release of DAMPs follows cell death due to secondary injury processes occurring later than the acute cytokine response. Given the relationship between NOD2 activation and extracranial injury, NF-κB activation may also be influenced by other general injury factors.

Interestingly, an association was found between acute alcohol intoxication and lower NOD2 activation in both the acute phase and at one-month post-mTBI. Previous clinical research has shown that acute alcohol intoxication is protective against subacute symptoms(Scheenen et al., 2015) and poor long-term functional outcome after mTBI (van der Naalt et al., 2017). Furthermore, in rats with hemorrhagic shock and resuscitation, a single dose of alcohol (30% solution, 5 g/kg) administered before experimental procedures reduces the serum pro-inflammatory response (including IL-6 release) via inhibition of NF-κB (Relja et al., 2012). Although acute alcohol intoxication may reduce inflammation following traumatic injury, alcohol does exert numerous negative effects throughout the body, including gut (promoting leakiness) and brain, mediated through inflammation (Anand et al., 2023). Therefore, we refrain from speculating about the potential clinical role of administration of a single alcohol dose to promote recovery in the acute phase after mTBI.

Null associations with long-term clinical outcomes may suggest that NOD2 activity is not a valuable measure to include in future prognostic modeling. Recovery after mTBI is a complex, multifaceted process involving both physiological and psychological factors, and cannot be explained by any single mechanism (van der Horn et al., 2020). Furthermore, this complex process likely varies across individuals, such that two patients with poor recovery may not share the exact same underlying pathophysiological mechanisms. Activation of NOD2 may be viewed as a broader measure of (neuro)inflammation, indirectly influencing recovery through (multiple) intermediary biochemical processes; therefore, its potential role in combined biomarker panels warrants further investigation. Interestingly, chronic stimulation of NOD2 by bacterial products may decrease the pro-inflammatory response, suggesting NOD2-mediated innate immune tolerance (Hedl et al., 2007). More research is needed on a broader spectrum of markers to determine the role of (pre- and post-injury) gut health in prognostic modeling of mTBI. Furthermore, given that aging is associated with increased inflammation (Franceschi et al., 2000), age may have contributed to our observed NOD2 effects; however, the persistence of large effect sizes when analyses were repeated with a younger age-matched mTBI subgroup suggests that the main effects are not solely driven by age.

Our study has several strengths and weaknesses. A strength of this study is the inclusion of a well-characterized ED mTBI cohort, with follow-up serum sampling and long-term clinical outcome measurements. Another strength is that additional measures of inflammation (i.e., IL-6 and IL-10) were included, although these are not fully specific to intracranial processes (neuroinflammation) and represent only a small subset rather than a comprehensive assessment of systemic inflammation. Complementary advanced imaging techniques, such as MRI spectroscopy, may provide more direct insight into neuroinflammatory mechanisms (Birg et al., 2024). While sensitivity analyses provided some reassurance, an important limitation is the relatively small and not age-matched HC group, which may have limited our ability to fully capture the natural variability in MDP concentrations and NOD2 activity. Since blood alcohol level measurements are not routinely performed at our hospital, we could not investigate any potential dose response relationships regarding NOD2 activation. Furthermore, no data regarding inter-assay variability was acquired. Lastly, our findings should be considered preliminary as we did not investigate other markers of gut permeability (e.g., LPS, fatty acid binding protein 2), gut microbiome composition, potential confounding factors for blood peptidoglycan fragment levels, such as diet, bowel habits, renal clearance, and medical comorbidities or treatments (e.g., antibiotic exposure) (Inczefi et al., 2022; Troha et al., 2019).

In conclusion, our work does not support disrupted gut barrier integrity in the acute phase following mTBI or at one-month follow-up. Further research with longitudinal sampling during the first month post-injury, and the inclusion of additional markers of intestinal epithelial integrity, is needed to determine whether mTBI leads to transient changes in gut barrier integrity in humans. Our findings reaffirm the role of inflammation in mTBI and show that NOD2-expressing cells may be useful to study chronic (neuro)inflammatory processes.

## CRediT authorship contribution statement

**Koen Visser:** Writing – review & editing, Writing – original draft, Visualization, Software, Investigation, Formal analysis, Data curation. **Yue Wang:** Writing – review & editing, Resources, Methodology, Investigation, Data curation, Conceptualization. **Jon D. Laman:** Writing – review & editing, Conceptualization. **Myrthe E. de Koning:** Writing – review & editing, Project administration, Data curation. **Xiaoli Xu:** Formal analysis, Investigation, Methodology. **Andrei A. Vakhtin:** Writing – review & editing. **Andrew R. Mayer:** Writing – review & editing. **Daan Kremer:** Writing – review & editing, Conceptualization. **Stephan J.L. Bakker:** Writing – review & editing, Resources, Conceptualization. **Harry van Goor:** Writing – review & editing. **Joukje van der Naalt:** Writing – review & editing. **Arno R. Bourgonje:** Writing – review & editing, Supervision, Conceptualization. **Harm J. van der Horn:** Writing – review & editing, Writing – original draft, Supervision, Project administration, Investigation, Funding acquisition, Formal analysis, Data curation, Conceptualization.

## Study funding

This research was supported by a Mandema stipend (reference number MA 18-02) supporting research by residents, from the 10.13039/501100005075University Medical Center Groningen, The Netherlands, to H.J.v.d.H.

## Declaration of competing interest

None of the authors have any competing interests to declare.

## Data Availability

The authors do not have permission to share data.

## References

[bib1] Aghakhani N. (2022). Relationship between mild traumatic brain injury and the gut microbiome: a scoping review. J. Neurosci. Res..

[bib2] Anand S.K., Ahmad M.H., Sahu M.R., Subba R., Mondal A.C. (2023). Detrimental effects of alcohol-induced inflammation on brain health: from neurogenesis to neurodegeneration. Cell. Mol. Neurobiol..

[bib3] Arrieta M.C., Bistritz L., Meddings J.B. (2006). Alterations in intestinal permeability. Gut.

[bib4] Baker S.P., O'Neill B., Haddon W., Long W.B. (1974). The injury severity score: a method for describing patients with multiple injuries and evaluating emergency care. J. Trauma.

[bib5] Birg A., van der Horn H.J., Ryman S.G., Branzoli F., Deelchand D.K., Quinn D.K., Mayer A.R., Lin H.C., Erhardt E.B., Caprihan A., Zotev V., Parada A.N., Wick T.V., Matos Y.L., Barnhart K.A., Nitschke S.R., Shaff N.A., Julio K.R., Prather H.E., Vakhtin A.A. (2024). Diffusion magnetic resonance spectroscopy captures microglial reactivity related to gut-derived systemic lipopolysaccharide: a preliminary study. Brain Behav. Immun..

[bib6] Braniste V., Al-Asmakh M., Kowal C., Anuar F., Abbaspour A., Tóth M., Korecka A., Bakocevic N., Guan N.L., Kundu P., Gulyás B., Halldin C., Hultenby K., Nilsson H., Hebert H., Volpe B.T., Diamond B., Pettersson S. (2014). The gut microbiota influences blood-brain barrier permeability in mice. Sci. Transl. Med..

[bib7] Cassidy J.D., Boyle E., Carroll L.J. (2014). Population-based, inception cohort study of the incidence, course, and prognosis of mild traumatic brain injury after motor vehicle collisions. Arch. Phys. Med. Rehabil..

[bib8] Cavassani K.A., Ishii M., Wen H., Schaller M.A., Lincoln P.M., Lukacs N.W., Hogaboam C.M., Kunkel S.L. (2008). TLR3 is an endogenous sensor of tissue necrosis during acute inflammatory events. J. Exp. Med..

[bib9] Chaban V., Clarke G.J.B., Skandsen T., Islam R., Einarsen C.E., Vik A., Damås J.K., Mollnes T.E., Håberg A.K., Pischke S.E. (2020). Systemic inflammation persists the first year after mild traumatic brain injury: results from the prospective trondheim mild traumatic brain injury study. J. Neurotrauma.

[bib10] Cuzzocrea S., Doyle T., Campolo M., Paterniti I., Esposito E., Farr S.A., Salvemini D. (2018). Sphingosine 1-Phosphate receptor subtype 1 as a therapeutic target for brain trauma. J. Neurotrauma.

[bib11] de Koning M.E., Gareb B., el Moumni M., Scheenen M.E., van der Horn H.J., Timmerman M.E., Spikman J.M., van der Naalt J. (2016). Subacute posttraumatic complaints and psychological distress in trauma patients with or without mild traumatic brain injury. Injury.

[bib12] Franceschi C., Bonafè M., Valensin S., Olivieri F., De Luca M., Ottaviani E., De Benedictis G. (2000). Inflamm-aging. An evolutionary perspective on immunosenescence. Ann. N. Y. Acad. Sci..

[bib13] Giza C.C., Hovda D.A. (2014). The new neurometabolic cascade of concussion. Neurosurgery.

[bib14] Hang C.H., Shi J.X., Li J.S., Wu W., Yin H.X. (2003). Alterations of intestinal mucosa structure and barrier function following traumatic brain injury in rats. World J. Gastroenterol..

[bib15] Hedl M., Li J., Cho J.H., Abraham C. (2007). Chronic stimulation of Nod2 mediates tolerance to bacterial products. Proc. Natl. Acad. Sci. U. S. A..

[bib16] Huang Z., Wang J., Xu X., Wang H., Qiao Y., Chu W.C., Xu S., Chai L., Cottier F., Pavelka N., Oosting M., Joosten L.A.B., Netea M., Ng C.Y.L., Leong K.P., Kundu P., Lam K.P., Pettersson S., Wang Y. (2019). Antibody neutralization of microbiota-derived circulating peptidoglycan dampens inflammation and ameliorates autoimmunity. Nat. Microbiol..

[bib17] Inczefi O., Bacsur P., Resál T., Keresztes C., Molnár T. (2022). The influence of nutrition on intestinal permeability and the microbiome in health and disease. Front. Nutr..

[bib18] Keestra-Gounder A.M., Byndloss M.X., Seyffert N., Young B.M., Chávez-Arroyo A., Tsai A.Y., Cevallos S.A., Winter M.G., Pham O.H., Tiffany C.R., De Jong M.F., Kerrinnes T., Ravindran R., Luciw P.A., McSorley S.J., Bäumler A.J., Tsolis R.M. (2016). NOD1 and NOD2 signalling links ER stress with inflammation. Nature.

[bib19] Kelly J.R., Kennedy P.J., Cryan J.F., Dinan T.G., Clarke G., Hyland N.P. (2015). Breaking down the barriers: the gut microbiome, intestinal permeability and stress-related psychiatric disorders. Front. Cell. Neurosci..

[bib20] Krämer T.J., Hübener P., Pöttker B., Gölz C., Neulen A., Pantel T., Goetz H., Ritter K., Schäfer M.K.E., Thal S.C. (2022). Ribonuclease-1 treatment after traumatic brain injury preserves blood–brain barrier integrity and delays secondary brain damage in mice. Sci. Rep..

[bib21] Laman J.D., ’t Hart B.A., Power C., Dziarski R. (2020). Bacterial peptidoglycan as a driver of chronic brain inflammation. Trends Mol. Med..

[bib22] Maas A.I.R., Menon D.K., Manley G.T., Abrams M., Åkerlund C., Andelic N., Aries M., Bashford T., Bell M.J., Bodien Y.G., Brett B.L., Büki A., Chesnut R.M., Citerio G., Clark D., Clasby B., Cooper D.J., Czeiter E., Czosnyka M., Dams-O’Connor K., De Keyser V., Diaz-Arrastia R., Ercole A., Van Essen T.A., Falvey E., Ferguson A.R., Figaji A., Fitzgerald M., Foreman B., Gantner D., Gao G., Giacino J., Gravesteijn B., Guiza F., Gupta D., Gurnell M., Haagsma J.A., Hammond F.M., Hawryluk G., Hutchinson P., Van Der Jagt M., Jain Sonia, Jain Swati, Jiang J.Y., Kent H., Kolias A., Kompanje E.J.O., Lecky F., Lingsma H.F., Maegele M., Majdan M., Markowitz A., McCrea M., Meyfroidt G., Mikolić A., Mondello S., Mukherjee P., Nelson D., Nelson L.D., Newcombe V., Okonkwo D., Orešič M., Peul W., Pisicǎ D., Polinder S., Ponsford J., Puybasset L., Raj R., Robba C., Røe C., Rosand J., Schueler P., Sharp D.J., Smielewski P., Stein M.B., Von Steinbuchel N., Stewart W., Steyerberg E.W., Stocchetti N., Temkin N., Tenovuo O., Theadom A., Thomas I., Espin A.T., Turgeon A.F., Unterberg A., Van Praag D., Van Veen E., Verheyden J., Vande Vyvere T., Wang K.K.W., Wiegers E.J.A., Williams W.H., Wilson L., Wisniewski S.R., Younsi A., Yue J.K., Yuh E.L., Zeiler F.A., Zeldovich M., Zemek R., Adams H., Agnoletti V., Allanson J., Amrein K., Andaluz N., Anke A., Antoni A., Van As A.B., Audibert G., Azaševac A., Azouvi P., Azzolini M.L., Baciu C., Badenes R., Barlow K.M., Bartels R., Bauerfeind U., Beauchamp M., Beer D., Beer R., Belda F.J., Bellander B.M., Bellier R., Benali H., Benard T., Beqiri V., Beretta L., Bernard F., Bertolini G., Bilotta F., Blaabjerg M., Den Boogert H., Boutis K., Bouzat P., Brooks B., Brorsson C., Bullinger M., Burns E., Calappi E., Cameron P., Carise E., Castaño-León A.M., Causin F., Chevallard G., Chieregato A., Christie B., Cnossen M., Coles J., Collett J., Della Corte F., Craig W., Csato G., Csomos A., Curry N., Dahyot-Fizelier C., Dawes H., DeMatteo C., Depreitere B., Dewey D., Van Dijck J., Dilvesi D., Dippel D., Dizdarevic K., Donoghue E., Duek O., Dulière G.L., Dzeko A., Eapen G., Emery C.A., English S., Esser P., Ezer E., Fabricius M., Feng J., Fergusson D., Fleming J., Foks K., Francony G., Freedman S., Freo U., Frisvold S.K., Gagnon I., Galanaud D., Giraud B., Glocker B., Golubovic J., López P.A.G., Gordon W.A., Gradisek P., Gravel J., Griesdale D., Grossi F., Håberg A.K., Haitsma I., Van Hecke W., Helbok R., Helseth E., Van Heugten C., Hoedemaekers C., Höfer S., Horton L., Hui J., Huijben J.A., Jacobs B., Jankowski S., Janssens K., Jelaca B., Jones K.M., Kamnitsas K., Kaps R., Karan M., Katila A., Kaukonen K.M., Kivisaari R., Kolumbán B., Kolundžija K., Kondziella D., Koskinen L.O., Kovács N., Kramer A., Kutsogiannis D., Kyprianou T., Lagares A., Lamontagne F., Latini R., Lauzier F., Lazar I., Ledig C., Lefering R., Legrand V., Levi L., Lightfoot R., Lozano A., MacDonald S., Major S., Manara A., Manhes P., Maréchal H., Martino C., Masala A., Masson S., Mattern J., McFadyen B., McMahon C., Meade M., Melegh B., Menovsky T., Moore L., Correia M.M., Morganti-Kossmann M.C., Muehlan H., Murray L., Van Der Naalt J., Negru A., Nieboer D., Noirhomme Q., Nyirádi J., Oddo M., Oldenbeuving A.W., Ortolano F., Osmond M., Payen J.F., Perlbarg V., Persona P., Pichon N., Piippo-Karjalainen A., Pili-Floury S., Pirinen M., Ple H., Poca M.A., Posti J., Ptito A., Radoi A., Ragauskas A., Real R.G.L., Reed N., Rhodes J., Robertson C., Rocka S., Røise O., Roks G., Rosenfeld J.V., Rosenlund C., Rosenthal G., Rossi S., Rueckert D., De Ruiter G.C.W., Sacchi M., Sahakian B.J., Sahuquillo J., Sakowitz O., Salvato G., Sánchez-Porras R., Sándor J., Sangha G., Schäfer N., Schmidt S., Schneider K.J., Schnyer D., Schöhl H., Schoonman G.G., Schou R.F., Sir Ö., Skandsen T., Smeets D., Sorinola A., Stamatakis E., Stevanovic A., Stevens R.D., Sundström N., Taccone F.S., Takala R., Tanskanen P., Taylor M.S., Telgmann R., Teodorani G., Thomas M., Tolias C.M., Trapani T., Vajkoczy P., Valadka A.B., Valeinis E., Vallance S., Vámos Z., Vargiolu A., Vega E., Vik A., Vilcinis R., Vleggeert-Lankamp C., Vogt L., Volovici V., Voormolen D.C., Vulekovic P., Van Waesberghe J., Wessels L., Wildschut E., Williams G., Winkler M.K.L., Wolf S., Wood G., Xirouchaki N., Zaaroor M., Zelinkova V., Zumbo F. (2022). Traumatic brain injury: progress and challenges in prevention, clinical care, and research. Lancet Neurol..

[bib23] Manley G.T., Dams-O’Connor K., Alosco M.L., Awwad H.O., Bazarian J.J., Bragge P., Corrigan J.D., Doperalski A., Ferguson A.R., Mac Donald C.L., Menon D.K., McNett M.M., van der Naalt J., Nelson L.D., Pisică D., Silverberg N.D., Umoh N., Wilson L., Yuh E.L., Zetterberg H., Maas A.I.R., McCrea M.A. (2025). A new characterisation of acute traumatic brain injury: the NIH-NINDS TBI classification and nomenclature initiative. Lancet Neurol..

[bib24] McMahon P., Hricik A., Yue J.K., Puccio A.M., Inoue T., Lingsma H.F., Beers S.R., Gordon W.A., Valadka A.B., Manley G.T., Okonkwo D.O., Casey S.S., Cooper S.R., Dams-O’Connor K., Menon D.K., Sorani M.D., Yuh E.L., Mukherjee P., Schnyer D.M., Vassar M.J. (2014). Symptomatology and functional outcome in mild traumatic brain injury: results from the prospective TRACK-TBI study. J. Neurotrauma.

[bib25] Mourits V.P., Koeken V.A.C.M., De Bree L.C.J., Moorlag S.J.C.F.M., Chu W.C., Xu X., Dijkstra H., Lemmers H., Joosten L.A.B., Wang Y., Van Crevel R., Netea M.G. (2020). BCG-induced trained immunity in healthy individuals: the effect of plasma muramyl dipeptide concentrations. J. Immunol. Res..

[bib26] Munley J.A., Kirkpatrick S.L., Gillies G.S., Bible L.E., Efron P.A., Nagpal R., Mohr A.M. (2023). The intestinal microbiome after traumatic injury. Microorganisms.

[bib27] Negroni A., Pierdomenico M., Cucchiara S., Stronati L. (2018). NOD2 and inflammation: current insights. J. Inflamm. Res..

[bib28] Nicholson S.E., Watts L.T., Burmeister D.M., Merrill D., Scroggins S., Zou Y., Lai Z., Grandhi R., Lewis A.M., Newton L.M., Eastridge B.J., Schwacha M.G. (2019). Moderate traumatic brain injury alters the gastrointestinal microbiome in a time-dependent manner. Shock.

[bib29] Olsen A.B., Hetz R.A., Xue H., Aroom K.R., Bhattarai D., Johnson E., Bedi S., Cox C.S., Uray K. (2013). Effects of traumatic brain injury on intestinal contractility. Neuro Gastroenterol. Motil..

[bib30] Oyovwi M.O., Udi O.A. (2024). The gut-brain axis and neuroinflammation in traumatic brain injury. Mol. Neurobiol..

[bib31] Pan P., Song Y., Du X., Bai L., Hua X., Xiao Y., Yu X. (2019). Intestinal barrier dysfunction following traumatic brain injury. Neurol. Sci..

[bib32] Panther E.J., Dodd W., Clark A., Lucke-Wold B. (2022). Gastrointestinal microbiome and neurologic injury. Biomedicines.

[bib33] Parker A., Fonseca S., Carding S.R. (2020). Gut microbes and metabolites as modulators of blood-brain barrier integrity and brain health. Gut Microbes.

[bib34] Pei G., Zyla J., He L., Moura‐Alves P., Steinle H., Saikali P., Lozza L., Nieuwenhuizen N., Weiner J., Mollenkopf H., Ellwanger K., Arnold C., Duan M., Dagil Y., Pashenkov M., Boneca I.G., Kufer T.A., Dorhoi A., Kaufmann S.H. (2021). Cellular stress promotes NOD1/2‐dependent inflammation via the endogenous metabolite sphingosine‐1‐phosphate. EMBO J..

[bib35] Petersen C., Round J.L. (2014). Defining dysbiosis and its influence on host immunity and disease. Cell. Microbiol..

[bib36] Relja B., Höhn C., Bormann F., Seyboth K., Henrich D., Marzi I., Lehnert M. (2012). Acute alcohol intoxication reduces mortality, inflammatory responses and hepatic injury after haemorrhage and resuscitation in vivo. Br. J. Pharmacol..

[bib37] Sage N. Le, Chauny J.M., Berthelot S., Archambault P., Neveu X., Moore L., Boucher V., Frenette J., De Guise É., Ouellet M.C., Lee J., Mcrae A.D., Lang E., Émond M., Mercier É., Tardif P.A., Swaine B., Cameron P., Perry J.J. (2022). Post-concussion symptoms rule: derivation and validation of a clinical decision rule for early prediction of persistent symptoms after a mild traumatic brain injury. J. Neurotrauma.

[bib38] Scheenen M.E., de Koning M.E., van der Horn H.J., Roks G., Yilmaz T., van der Naalt J., Spikman J.M. (2015). Acute alcohol intoxication in patients with mild traumatic brain injury: characteristics, recovery, and outcome. J. Neurotrauma.

[bib39] Shi H., Hua X., Kong D., Stein D., Hua F. (2019). Role of toll-like receptor mediated signaling in traumatic brain injury. Neuropharmacology.

[bib40] Soriano S., Curry K., Sadrameli S.S., Wang Q., Nute M., Reeves E., Kabir R., Wiese J., Criswell A., Schodrof S., Britz G.W., Gadhia R., Podell K., Treangen T., Villapol S. (2022). Alterations to the gut microbiome after sport-related concussion in a collegiate football players cohort: a pilot study. Brain Behav. Immun. Health.

[bib41] Spielbauer J., Glotfelty E.J., Sarlus H., Harris R.A., Diaz Heijtz R., Karlsson T.E. (2024). Bacterial peptidoglycan signalling in microglia: activation by MDP via the NF-κB/MAPK pathway. Brain Behav. Immun..

[bib42] Sundman M.H., Chen N. kuei, Subbian V., Chou Y. hui (2017). The bidirectional gut-brain-microbiota axis as a potential nexus between traumatic brain injury, inflammation, and disease. Brain Behav. Immun..

[bib43] Tan C.T., Xu X., Qiao Y., Wang Y. (2021). A peptidoglycan storm caused by β-lactam antibiotic's action on host microbiota drives Candida albicans infection. Nat. Commun..

[bib44] Troha K., Nagy P., Pivovar A., Lazzaro B.P., Hartley P.S., Buchon N. (2019). Nephrocytes remove microbiota-derived peptidoglycan from systemic circulation to maintain immune homeostasis. Immunity.

[bib45] van der Horn H.J., Out M.L., de Koning M.E., Mayer A.R., Spikman J.M., Sommer I.E., van der Naalt J. (2020). An integrated perspective linking physiological and psychological consequences of mild traumatic brain injury. J. Neurol..

[bib46] van der Naalt J., Timmerman M.E., de Koning M.E., van der Horn H.J., Scheenen M.E., Jacobs B., Hageman G., Yilmaz T., Roks G., Spikman J.M. (2017). Early predictors of outcome after mild traumatic brain injury (UPFRONT): an observational cohort study. Lancet Neurol..

[bib47] Visser K., Ciubotariu D., de Koning M.E., Jacobs B., van Faassen M., van der Ley C., Mayer A.R., Meier T.B., Bourgonje A.R., Kema I.P., van Goor H., van der Naalt J., van der Horn H.J. (2024). Exploring the kynurenine pathway in mild traumatic brain injury: a longitudinal study. J. Neurochem..

[bib48] Visser K., de Koning M.E., Ciubotariu D., Kok M.G.J., Sibeijn-Kuiper A.J., Bourgonje A.R., van Goor H., van der Naalt J., van der Horn H.J. (2024). An exploratory study on the association between blood-based biomarkers and subacute neurometabolic changes following mild traumatic brain injury. J. Neurol..

[bib49] Visser K., Koggel M., Blaauw J., van der Horn H.J., Jacobs B., van der Naalt J. (2022). Blood-based biomarkers of inflammation in mild traumatic brain injury: a systematic review. Neurosci. Biobehav. Rev..

[bib50] Vourc’h M., Roquilly A., Asehnoune K. (2018). Trauma-induced damage-associated molecular patterns-mediated remote organ injury and immunosuppression in the acutely Ill patient. Front. Immunol..

[bib51] Wilson L., Boase K., Nelson L.D., Temkin N.R., Giacino J.T., Markowitz A.J., Maas A., Menon D.K., Teasdale G., Manley G.T. (2021). A manual for the Glasgow outcome scale-extended interview. J. Neurotrauma.

[bib52] Zhao J., Bi W., Xiao S., Lan X., Cheng X., Zhang J., Lu D., Wei W., Wang Y., Li H., Fu Y., Zhu L. (2019). Neuroinflammation induced by lipopolysaccharide causes cognitive impairment in mice. Sci. Rep..

