# Supplementary material for: Stable serum peptidoglycan fragment levels do not support leaky gut in the acute phase or at one month following “mild” traumatic brain injury: A preliminary study
Source: Brain Behav Immun Health. 2026 Feb 5;52:101192. doi: 10.1016/j.bbih.2026.101192 (PMC12908069; doi:10.1016/j.bbih.2026.101192)
Supplement: Multimedia component 1 [file mmc1.docx]

*Supplementary Materials for:*

**Stable Serum Peptidoglycan Fragment Levels Do Not Support Leaky Gut in the Acute Phase or at One Month Following “Mild” Traumatic Brain Injury: A Preliminary Study**

**Methods**

*MDP assay details*

A coating solution (CS) was prepared by diluting human serum albumin (HSA)–MDP (HSA-MDP) in coating buffer (CB; 1.59g Na_2_CO_3_, 2.93g NaHCO_3_ in 1 L distilled water, pH=9.6) to obtain a final concentration of 2ug/mL. Then, 50 µL of CS was added to each well of a 96-well half-area flat-bottom ELISA plate followed by incubation at 4°C for 16-18 hours. The next day, contents of the wells were discarded, and plates were blotted dry. Then 50 µL of blocking solution (1% gelatin from cold water fish skin in CS) was added to each well and the plate was incubated at room temperature (RT) for 1 hour on an orbital shaker at 100 rpm. After blocking, contents of the wells were discarded, and plates were blotted dry. Then, wells were washed three times with 100 µL of wash buffer (1× PBST containing 0.05% Tween-20, pH 7.2–7.4), with a 1.5-minute incubation for each wash.
 All serum samples were diluted 2-fold with antibody dilution buffer (ADB; PBS containing 0.05% Tween-20 and 0.1% gelatin, pH=7.2-7.4). Fresh primary antibody (2E7; 1 mg/mL stock solution) was prepared by diluting it 1:150000 in ADB to a final concentration of 6.67 ng/mL. Then, 90 µL of the diluted 2E7 and 30 µL serum sample were added to each well of the 96-well V-bottom plates, and the plates were subsequently incubated for 1 hour at RT. Next, 50 µL from each well of the V-bottom plates was transferred to the corresponding wells of the flat-bottom ELISA plates. The transfer was performed twice to generate duplicates. Plates were incubated for 2 hours at room temperature (RT) on an orbital shaker at 100 rpm. After incubation, the contents of the wells were discarded, and plates were blotted dry. Wells were then washed three times with 100 µL of wash buffer, with a 1.5-minute incubation for each wash. After preparing the secondary antibody (HRP-conjugated goat anti-mouse IgG) in ADB at a 1:2,000 dilution, 50 µL was added to each well followed by incubation for 1 hour at room temperature (RT). The contents of the wells were then discarded, and plates were blotted dry. Subsequently, the wells were washed three times with 100 µL of wash buffer, with a 1.5-minute incubation for each wash, followed by a final wash using 200 µL of wash buffer. Then, 50 µL of TMB (3,3ʹ,5,5ʹ-Tetramethylbenzidine; Abcam, AB171527) substrate solution was added to each well and plates were incubated in the dark for 10 minutes at 37 °C. The reaction was stopped by adding 50 µL of stop solution (2 M H₂SO₄) and absorbance was measured at 450 nm using a plate reader.

*Sensitivity analyses*

MDP concentrations and NOD2 activity in the acute phase were compared between patients who returned for follow-up at ~1-month post-injury (N = 136) and those who did not (N = 106). For MDP, a general linear model was used with group (mild traumatic brain injury [mTBI] vs healthy controls [HC]) and age and sex as covariates. For NOD2, a linear mixed-effects model was applied with the same variables as fixed effects and plate included as a random effect. The same analytical approach was used to compare patients who did and did not complete the Glasgow Outcome Scale-Extended (GOS-E) and Head Injury Symptom Checklist (HISC) questionnaires.
 Given the significant age difference between the mTBI and HC groups, additional sensitivity analyses were conducted to assess the influence of age on the results. A subset of patients (N = 31) was selected from the full mTBI sample to optimally match the HC group (age: *P* = 0.98; sex: *P* = 0.80). The primary linear mixed models were then repeated to compare MDP concentrations and NOD2 activity between these matched groups.

**Results**
*Relationship between MDP concentrations and NOD2 activity*

In both the acute phase and at ~1-month follow-up, there was no significant rank correlation between MDP concentrations and NOD2 activity in the mTBI group (ρ_acute_=0.04, *P*=0.56; ρ_subacute_=0.11, *P*=0.20) or in the HC group (ρ_acute_=0.03, *P*=0.88; ρ_subacute_=0.16, *P*=0.43).

**Table S1.** Multivariate regression analyses examining the associations of acute and ~1-month nucleotide-binding oligomerization domain-containing protein 2 (NOD2) activity with injury and trauma care characteristics.

|  | **NOD2 (acute)** | | **NOD2 (~1-month)** | |
| --- | --- | --- | --- | --- |
|  | β* | *P* | β* | *P* |
| Time to sample (blood draw) | 0.00 | 0.95 | -0.06 | 0.44 |
| Presence of extracranial injury | -0.15 | 0.23 | 0.39 | **0.01** |
| Alcohol intoxication | -0.30 | **0.046** | -0.39 | **0.03** |
| Presence of lesions on head CT | -0.09 | 0.55 | -0.09 | 0.63 |
| GCS | 0.01 | 0.92 | 0.07 | 0.38 |

***Standardized β, one unit change represents one standard deviation.
Uncorrected *P*-values significant at α<0.05 are listed in bold face type.

*Abbreviations:* CT = computed tomography; GCS = Glasgow Coma Scale; NOD2 = nucleotide-binding oligomerization domain-containing protein 2; TBI = traumatic brain injury.

**Table S2.** Multivariate regression analyses examining the associations of acute and ~1-month muramyl dipeptide (MDP) concentrations with acute injury and trauma care characteristics.

|  | **MDP (acute)** | | **MDP (~1-month)** | |
| --- | --- | --- | --- | --- |
|  | β* | *P* | β* | *P* |
| Time to sample (blood draw) | -0.05 | 0.47 | -0.08 | 0.42 |
| Presence of extracranial injury | -0.10 | 0.44 | -0.13 | 0.50 |
| Alcohol intoxication | -0.15 | 0.35 | -0.03 | 0.89 |
| Presence of lesions on head CT | -0.06 | 0.71 | 0.16 | 0.47 |
| GCS | -0.00 | 1.0 | -0.05 | 0.57 |

***Standardized β, one unit change represents one standard deviation.

*Abbreviations:* CT = computed tomography; GCS = Glasgow Coma Scale; MDP = muramyl dipeptide; TBI = traumatic brain injury.
